# Lanthanide-Doped Upconversion Nanocarriers for Drug and Gene Delivery

**DOI:** 10.3390/nano8070511

**Published:** 2018-07-09

**Authors:** Gibok Lee, Yong Il Park

**Affiliations:** School of Chemical Engineering, Chonnam National University, Gwangju 61186, Korea; 188608@jnu.ac.kr

**Keywords:** upconversion nanoparticles, nanocarriers, drug delivery, gene delivery, photolysis

## Abstract

Compared to traditional cancer treatments, drug/gene delivery is an advanced, safe, and efficient method. Nanoparticles are widely used as nanocarriers in a drug/gene delivery system due to their long circulation time and low multi-drug resistance. In particular, lanthanide-doped upconversion nanoparticles (UCNPs) that can emit UV and visible light by near-infrared (NIR) upconversion demonstrated more efficient and safer drug/gene delivery. Because of the low penetration depth of UV and visible light, a photoinduced reaction such as photocleavage or photoisomerization has proven restrictive. However, NIR light has high tissue penetration depth and stimulates the photoinduced reaction through UV and visible emissions from lanthanide-doped UCNPs. This review discusses the optical properties of UCNPs that are useful in bioapplications and drug/gene delivery systems using the UCNPs as a photoreaction inducer.

## 1. Introduction

Cancer is one of the major diseases that threatens human health worldwide. Many researchers have developed various cancer treatments such as surgery, chemotherapy, and radiotherapy. However, these methods have limitations. Because cancer lesions and normal tissue are not completely differentiated during surgery, large complex tissues are removed. Chemotherapy works on the whole body in addition to the cancerous tissues and results in low tumor-specific targeting, severe side effects, drug dependence, and multi-drug resistance (MDR) [1,2,3,4]. Radiotherapy works on a specific region with normal tissues and results in skin damage, cancer recurrence, cell mutation, dose-dependent side effects, and cardiovascular problems [5,6,7]. Therefore, the development of more efficient and safe methods to remove cancerous tissues is essential for improving cancer treatments. Recently, drug/gene delivery systems have been suggested as an alternative to conventional cancer treatments. In particular, nanoparticles used as drug/gene carriers are attracting attention due to advantages such as their high tumor-specific targeting with low side effects and increased loading capacity. Nanoparticles tend to accumulate in tumor lesions through the relatively leaky tumor vasculature, and this accumulation is associated with the enhanced permeability and retention (EPR) effect [8,9,10,11]. The large specific surface area of nanoparticles and mesoporous/hollow nanostructures increases the amount of drug/gene loading. To exploit these advantages, many researchers have designed nanocarriers such as liposomes [12,13,14,15], polymeric micelles [16,17,18], polymeric nanoparticles [19,20,21], carbon nanotubes [22,23], reduced graphene oxides [24,25,26], gold nanoparticles [27,28,29], magnetic nanoparticles [30,31,32], and lanthanide-doped upconversion nanoparticles (UCNPs) [33,34,35,36,37,38].

In this review, we discuss drug/gene delivery systems that use lanthanide-doped UCNPs. The unique and fascinating optical properties that convert near-infrared (NIR) light to visible and UV light allow UCNPs to be used as more efficient nanocarrier materials in drug/gene delivery systems. This drug/gene delivery system is based on photoinduced reactions such as photocleavage and photoisomerization. Photoinduced delivery systems enhance the efficacy of spatial and temporal control of drug/gene release and minimize normal cell death, side effects, and the tissue damage.

## 2. Lanthanide-Doped UCNPs

**Figure 1:**
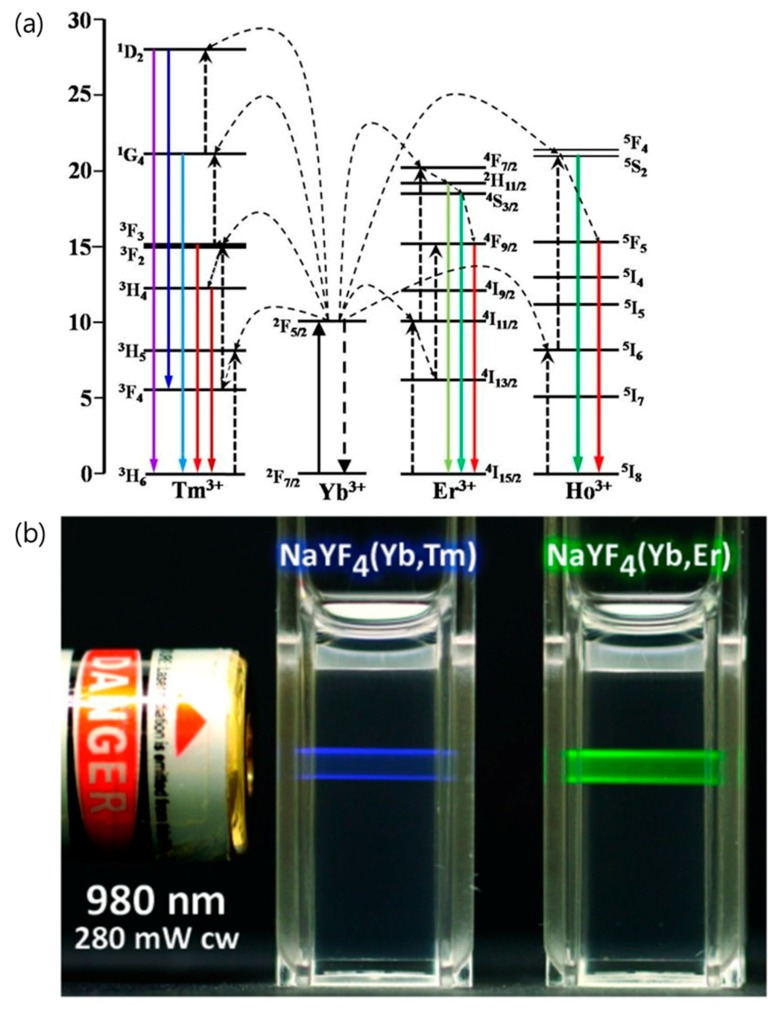
(**a**) Energy level diagrams of Tm^3+^, Yb^3+^, Er^3+^, and Ho^3+^ ions, illustrating the upconversion mechanism in lanthanide-doped upconversion nanoparticles (UCNPs) [43]. (**b**) Photographs of upconversion emission excited by a 980 nm laser [51]. Reproduced with permission from [43]. Copyright MDPI, 2017. Reproduced with permission from [51]. Copyright IVYSPRING, 2013.

Photoinduced drug/gene delivery systems use photoreactions to control drug release and gene expression, mainly through UV light sources. However, the UV region is not only harmful to tissues and cells, but also has a disadvantage of low tissue penetration depth due to its absorption by biological substances (e.g., protein, hemoglobin, and melanin) [39,40]. Unlike UV light, NIR light exhibit the deep penetration depth due to low absorption by biological substances and minimizes photodamage to tissues and cells [39,41]. In order to use NIR light for photoinduced drug/gene delivery, an anti-Stokes shifting material that emits UV light by NIR excitation is required. Among the various materials, UCNPs have been proposed as a strong candidate. Upconversion is the process by which multiple lower energy photons are converted to higher energy photons and are emitted [42]. Various lanthanide-doped UCNPs have been extensively investigated, and ytterbium (Yb^3+^), erbium (Er^3+^), thulium (Tm^3+^), and holmium (Ho^3+^) are well-known lanthanide dopants in UCNPs (see Figure 1a) [43]. Yb^3+^ ions are usually used as a sensitizer for absorbing NIR photons, and Er^3+^, Tm^3+^, and Ho^3+^ ions are used as an activator to emit upconverted luminescence (i.e., visible and UV). In addition, because lanthanide ions produce long-lived luminescence emission through the forbidden 4f-4f transition between real intermediate energy states, upconversion is much more efficient than other anti-Stokes shift phenomena (e.g., two-photon absorption and second harmonic generation). Moreover, compact and inexpensive continuous wave (CW) lasers can be used as an excitation source for upconversion, while other anti-Stokes shifting materials require high power pulsed lasers [44,45]. Since energy transfer between the energy levels of lanthanide ions is possible, the choice of UCNP host material doped with lanthanide elements has a significant effect on the upconversion mechanism and its efficiency. The most widely used host material for lanthanide-mediated upconversion is sodium yttrium fluoride (NaYF_4_) because of its relatively low phonon energy (400 cm^−1^), which minimizes non-radiative relaxation decay [46,47]. Yb^3+^ and Er^3+^ co-doped UCNPs are primarily used as probes for in vitro and in vivo bioimaging [48,49,50]. The ^2^H_11/2_, ^4^S_3/2_ → ^4^I_15/2_, and ^4^F_9/2_ → ^4^I_15/2_ transitions in trivalent Er^3+^ emit the green and red upconverted luminescence (see Figure 1b) [51]. Green emission from Yb^3+^ and Er^3+^ co-doped UCNPs shows relatively high quantum yield compared to UCNPs with other lanthanide elements [52,53,54]. Yb^3+^ and Tm^3+^ co-doped UCNPs are primarily used as UV sources for photoinduced reactions. The ^1^I_6_ → ^3^H_6_, ^1^D_2_ → ^3^H_6_, ^1^D_2_ → ^3^F_4_, and ^1^G_4_ → ^3^H_6_ transition in trivalent Tm^3+^ emit the UV and blue upconverted luminescence (see Figure 1b) [51]. Thus, Yb^3+^ and Tm^3+^ co-doped UCNPs can induce photocleavage and photoisomerization of photosensitive compounds for drug/gene delivery systems.

**Figure 2:**
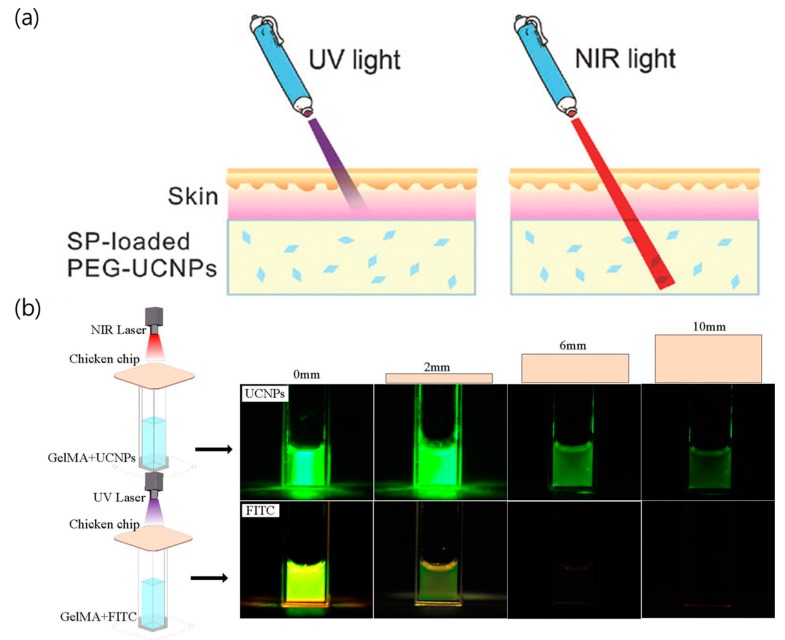
(**a**) Illustration of the penetration depths for UV and NIR light [55]. (**b**) Photographs of tissue penetration for NIR and UV light. Slices of chicken breast with various thicknesses (2 mm–10 mm) were placed between the laser source and the glass cuvette containing the UCNP-labeled hydrogel and fluorescein (FITC)-labeled hydrogel, respectively [56]. Reproduced with permission from [55]. Copyright The Royal Society of Chemistry, 2015. Reproduced with permission from [56]. Copyright Elsevier, 2017.

Figure 2a shows the difference in the penetration depths of UV and NIR light through pork skin placed on top of a solution containing UCNPs [55]. Figure 2b shows the penetration difference between UV and NIR lasers used to excite the fluorescein (FITC) and UCNPs, respectively. Under UV irradiation, the emission intensity from FITC decreased when the thickness of chicken breast increased, but the fluorescence signal from FITC was not observed when the thickness of chicken breast reached 6 mm. Under NIR irradiation, however, enough photons are transmitted to the UCNPs to emit green luminescence even though the thickness of the chicken is 10 mm [56]. Also, the penetration depth of NIR light for upconversion imaging was demonstrated to be 1.25 inches (3.2 cm) at the pork tissue [57]. Therefore, UCNPs can be used as effective UV sources that can cause photoinduced reactions under NIR irradiation with high penetration depth and can be used as nanocarriers for drug/gene delivery.

## 3. Drug/Gene Delivery Using Photocleavage

In drug/gene delivery, the photocleavage reaction of small molecules or polymer backbones is an attractive method to trigger drug release and gene expression. The photocleavage reaction has three methods, namely (1) the direct cleavage of the bond between the molecule and the carrier [37,58,59,60]; (2) a change in the charge on the carrier surface used to induce electrostatic repulsion between the molecules and the carrier [61,62]; and (3) the destruction of the carrier itself [63].

### 3.1. Direct Cleavage of the Bond between the Molecule and the Carrier

**Figure 3:**
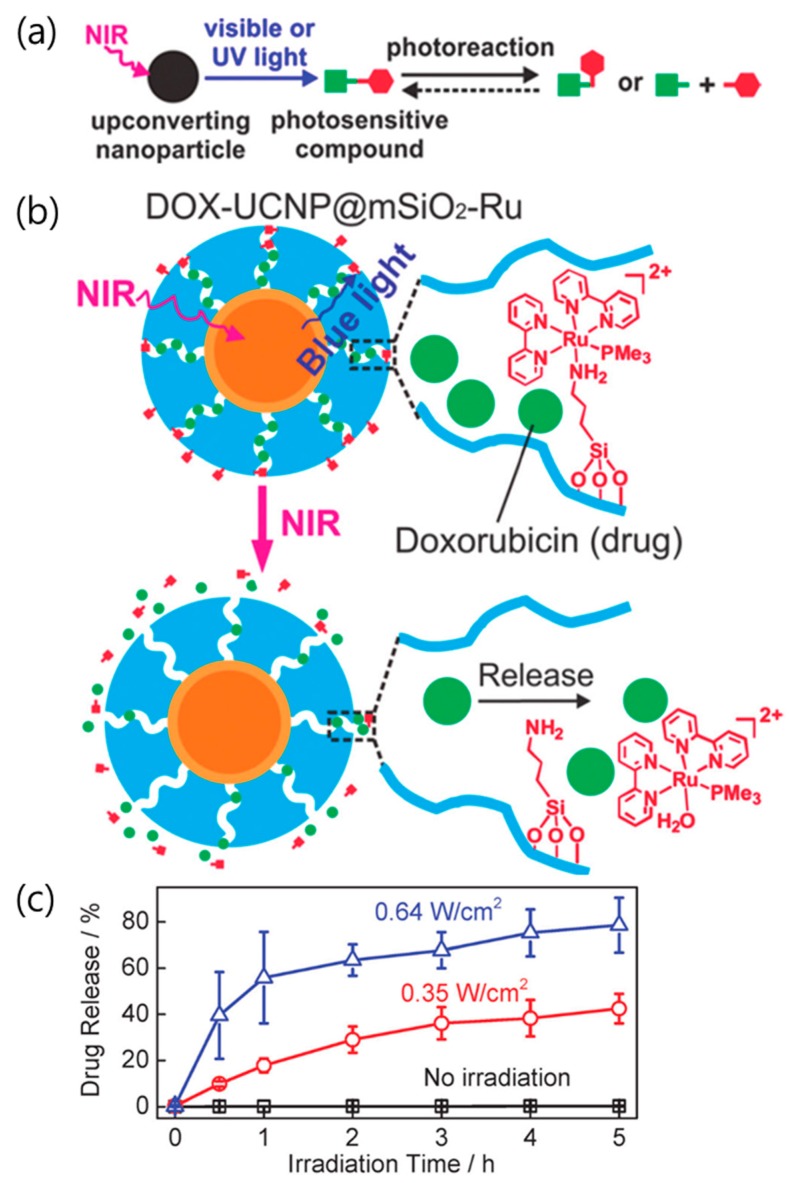
(**a**) Schematic illustration of photoreactions involving photosensitive compounds induced by UV and visible light produced by upconverted NIR radiation. (**b**) Under NIR irradiation, upconverted blue light triggers cleavage of the Ru complexes and drug release. (**c**) Doxorubicin release profiles with laser power and irradiation time. Reproduced with permission from [70]. Copyright The Royal Society of Chemistry, 2015.

The photosensitive compounds are modified to bind the drug/gene to the carrier through a variety of molecular interactions, thus undergoing irreversible deformation [37,58,60,64,65]. The most commonly used photosensitive molecules are coumarinyl and *o*-nitrobenzyl esters. They serve as linkers that bind the drug/gene to the carriers and are broken when exposed to UV or visible light. These photoreactive molecules can also be directly destroyed by two-photon absorption of NIR light [66,67]. However, the molecules have a small two-photon absorption cross-section and slow reaction rate [68]. Thus, this photoreaction involving two-photon absorption requires a higher NIR laser power and longer reaction time than a UV (or visible) source. Due to the NIR excitation and efficient upconverted UV and visible light emission, the UCNPs can be used to induce photocleavable reactions using NIR light. When combined with the high penetration depth of NIR light, the low energy requirement of the UCNPs, which can be used with inexpensive CW lasers instead of high energy pulsed lasers, also has the advantage of increasing the delivery efficiency [69]. In 2015, He et al. reported that the UCNPs could induce photoreactive drug delivery under ultralow-intensity NIR irradiation [70]. The authors used the photocleavable reaction of the Ru complex to release the drug from the carrier (see Figure 3a). Since the absorption (~453 nm) of the Ru complex overlaps with the blue emission from the UCNPs, the upconverted light by NIR excitation induces photocleavage of the Ru complex [71]. The mesoporous silica shell of the UCNPs (UCNP@mSiO_2_) was used as the drug carrier, and the Ru complex was attached to the pores to serve as a molecular valve. Without NIR irradiation, the release of doxorubicin from UCNP@mSiO_2_ was not detected. When irradiated with NIR light, doxorubicin clogged with the Ru complex in the UCNP@mSiO_2_ was released due to photocleavage of the Ru complex (see Figure 3b). Irradiation with 0.35 W cm^−2^ laser intensity was sufficient to trigger the UCNPs-assisted photoreaction and release doxorubicin. Figure 3c also shows how the drug release efficiency depends on the NIR laser output intensity. Approximately 78% of the doxorubicin was released under irradiation with 0.64 W cm^−2^ for 5 h, while ~42% of the doxorubicin was released under irradiation with 0.35 W cm^−2^ for 5 h. Direct cleavage of the photosensitive compound for the drug release is dependent on the irradiated laser power and irradiation time.

### 3.2. Change in the Charge on the Carrier Surface To Induce Electrostatic Repulsion

**Figure 4:**
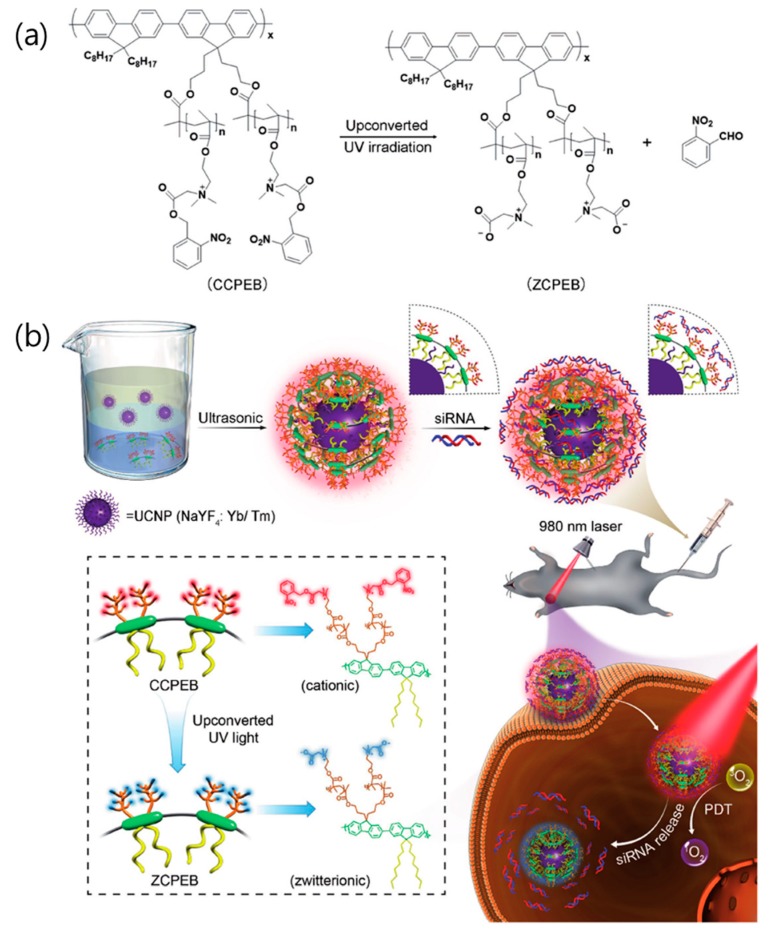
(**a**) Photoinduced charge-variable cationic conjugated polyelectrolyte brush (CCPEB). (**b**) Schematic representation of the NIR-induced charge-variable nanotherapeutic system (UCNP@CCPEB) for siRNA delivery and PDT. Reproduced with permission from [61]. Copyright John Wiley and Sons, 2017.

The delivery of chemical drugs enables effective therapy whether the carriers internalize into a cell or not. Contrary to chemical drug delivery, the carriers containing gene expression molecules must be internalized into a cell for gene therapy [72,73]. In addition, if the payload leakage is severe, the efficacy of gene therapy is greatly reduced. Therefore, the size and surface charge of the nanocarriers are important for cellular uptake. In particular, the positively charged surface of the nanocarriers demonstrates the increased efficiency of gene loading as well as an increased binding capacity to the anionic plasma membranes. The Fan group reported NIR-induced charge-variable cationic conjugated polyelectrolyte brushes (CCPEB) that encapsulate UCNPs for promoted siRNA release and cooperative photodynamic therapy (PDT) [61]. The charge-variable cationic conjugated polyelectrolyte (CCPE) was synthesized based on the *o*-nitrobenzyl ester and changed the surface charge on the carrier via the photocleavage reaction (see Figure 4a). CCPE has many positively charged quaternary amine groups that act as negatively charged nucleic acid carriers. The release of *o*-nitrobenzyl aldehyde by NIR irradiation converts the CCPEB on the UCNPs to zwitterionic conjugated polyelectrolyte brushes (ZCPEB) and initiates siRNA release by charge repulsion (see Figure 4b). As a result, UCNP@CCPEB as an siRNA carrier promoted siRNA release (80% efficiency) at pH 5.0 under 980 nm irradiation.

### 3.3. Destruction of the Carrier

**Figure 5:**
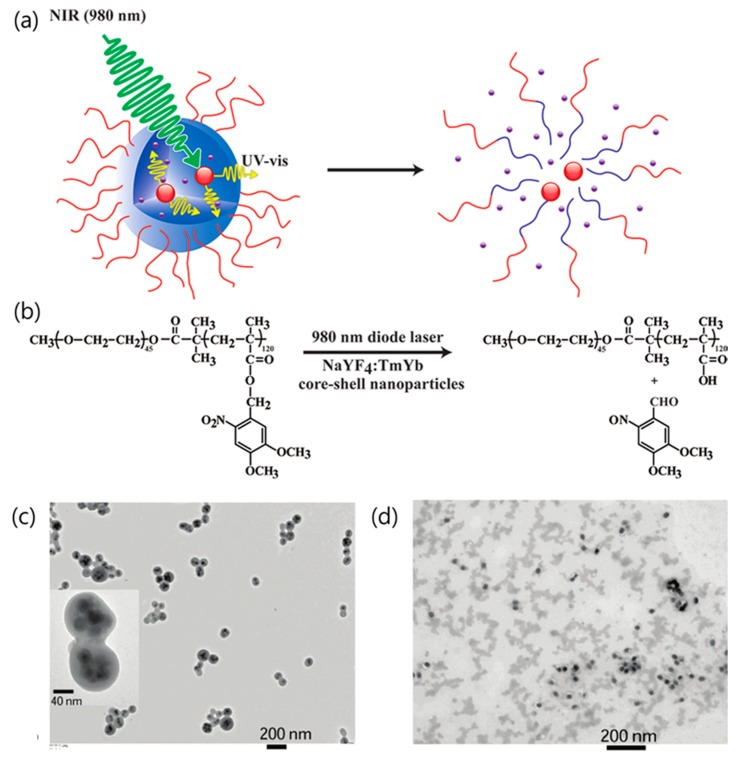
(**a**) Schematic of drug release resulting from NIR light-triggered dissociation of UCNP-loaded block copolymer (BCP) micelles. (**b**) Photoreaction scheme showing cleavage of the BCP backbone by NIR irradiation. (**c**,**d**) TEM images of the UCNP-loaded BCP micelles (**c**) before and (**d**) after NIR light irradiation. Reproduced with permission from [63]. Copyright American Chemical Society, 2011.

The nanocomposites encapsulating the drug molecules and functionalized nanomaterials increase the payload for effective chemotherapy. This strategy has the advantage of creating multifunctional materials by adding nanomaterials with various properties to the inside of the carrier. For example, UV emission from the UCNPs can destroy the drug carrier itself. In 2011, Yan et al. reported NIR light-triggered dissociation of block copolymer (BCP) micelles containing hydrophobic drugs and UCNPs (see Figure 5a) [63]. When the UCNPs in the BCP micelle were irradiated with NIR light, the polymethacrylate block in the BCP micelle was converted to hydrophilic poly-(methacrylic acid) by photocleavage of the hydrophobic *o*-nitrobenzyl groups (see Figure 5b). This collapse of the hydrophilic-hydrophobic balance destabilizes the BCP micelles [67]. Dissociation of the BCP micelles was also confirmed using TEM analysis. Before NIR irradiation, the BCP micelles contained 4-5 UCNPs per micelle (see Figure 5c). After excitation at 980 nm, the BCP micelles completely collapsed, and the UCNPs and drugs were released from the micelles (see Figure 5d).

## 4. Drug/Gene Delivery Using Photoisomerization

**Figure 6:**
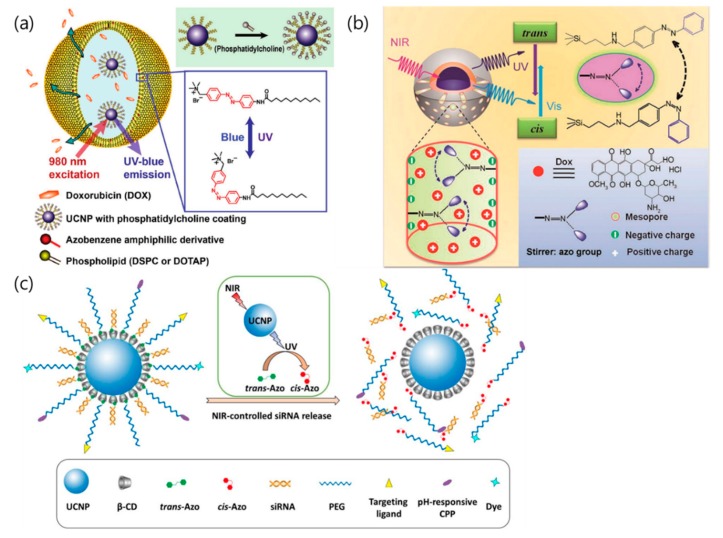
(**a**) Schematic illustration showing NIR light-triggered drug release of azobenzene-liposome/UCNPs hybrid vesicles by *trans*-*cis* photoisomerization [79]. (**b**) NIR-activated drug release from azo molecules grafted on mesopores by *trans*-*cis* photoisomerization [80]. (**c**) Illustration of NIR-triggered *trans*-to-*cis* photoisomerization of azobenzene, which subsequently leads to siRNA release from the UCNP-(CD/Azo)-siRNA [81]. Reproduced with permission from [79]. Copyright John Wiley and Sons, 2016. Reproduced with permission from [80]. Copyright John Wiley and Sons, 2013. Reproduced with permission from [81]. Copyright Elsevier, 2018.

Photoisomerization is a reaction in which isomers change their spatial conformation under optical irradiation [74,75,76,77]. Molecules such as azobenzene, spiropyran, and dithienylethene are mainly used, and their spatial conformation changes reversibly under UV and visible illumination [67,78]. This reaction can be used as a switch to control the drug/gene release by opening the pathway through which the drug/gene passes. In particular, azobenzene with a *trans*-*cis* transformation is mainly used for lanthanide-doped UCNPs [79,80,81,82,83]. In 2016, Yao et al. developed azobenzene-liposome/UCNPs hybrid vesicles for controlled drug delivery to overcome cancer MDR [79]. Azobenzene liposomes (Azo-Lipo) consists of 1,2-distearoyl-sn-glycero-3-phosphocholine (DSPC) phospholipids and azobenzene amphiphilic derivatives. The as-synthesized hydrophobic UCNPs (NaGdF_4_:Yb,Tm@NaGdF_4_) were encapsulated by DSPC phospholipids via van der Waals interaction to induce a hydrophilic surface [84]. The hydrophilic UCNPs and doxorubicin are located in the hydrophilic cavity of the vesicles along with formation of the doxorubicin-loaded Azo-Lipo/UCNPs hybrid vesicles. UV and visible light from the UCNPs enable reversible photoisomerization of the azobenzene derivatives. Continuous rotation-inversion motion by reversible *trans*-*cis* conversion destabilizes the lipid bilayer and releases the drug (see Figure 6a). This nanocomposite showed increased drug release efficiency with higher laser power and increased concentration of azobenzene derivatives. The release of doxorubicin reached 57 wt % in 6 h under intermittent 2.2 W cm^−2^ NIR laser irradiation, while over 90 wt % release can be reached in 6 h under intermittent 7.8 W cm^−2^ irradiation. The *trans*-*cis* transformation of azobenzene for drug release was also adapted to the mesopore structure on the UCNPs. In 2013, Liu et al. reported NIR-triggered anticancer drug delivery by UCNPs with an integrated azobenzene-modified mesoporous silica shell (Dox-UCNP@mSiO_2_-azo) [80]. The back and forth wagging motion of the azobenzene groups through *trans*-*cis* isomerization under UV and visible illumination acts as a molecular impeller to propel the release of doxorubicin from silica mesopores (see Figure 6b). Because the azobenzene molecular impeller is activated by light emitted from the UCNPs, drug release also depends on the laser power and duration. More doxorubicin was released with higher NIR illumination intensity. Conventional mesoporous silica releases the loaded drug by diffusion, the azobenzene impeller, regulated by the NIR light, controls the drug release more precisely. The azobenzene *trans*-*cis* transformation property has also been studied for use in gene delivery [85,86,87,88]. In 2017, Chen et al. reported an NIR-induced UCNP-based siRNA nanocarrier that exhibited spatiotemporally controlled gene silencing [81]. The UCNPs functionalized with β-cyclodextrin (CD) form the (UCNP-(CD/Azo)-siRNA) complex with siRNA-azobenzene through a host–guest interaction. *Trans*-azobenzene derivatives have a strong host-guest interaction with CD [77,89]. Under NIR irradiation, UV emission from the UCNPs induces photoisomerization of Azo-siRNAs into the cis configuration. The isomerized cis-azobenzene derivatives exhibit polarity change and steric hindrance, which destabilizes the host–guest interaction and releases siRNA (see Figure 6c). The amount of released siRNA can be easily controlled by selecting an appropriate NIR irradiation time. About 41% and 85% of siRNA was released within 10 and 20 min under 0.75 W cm^−2^ NIR irradiation, respectively. In addition, spatial gene silencing was controlled by spatial irradiation in the cell culture dish. Cells which are half-covered by aluminum foil and irradiated with the NIR laser (0.75 W cm^−2^, 10 min) show region-specific down-regulation of green fluorescent proteins. This controlled drug/gene delivery shows that the NIR-activated UCNPs are safer and more efficient carriers.

## 5. Conclusions and Future Prospects

The optical properties of lanthanide-doped UCNPs, which emit UV and visible light under NIR excitation, are useful for biomedical applications such as bioimaging and drug/gene delivery. In drug/gene delivery systems, UCNPs act as a source of UV light that cleaves the chemical link between the drug/gene and the nanocarrier and induces the photoisomerization of photosensitive compounds. The photocleavage of the photosensitive compound occurs by the direct cleavage of conjugation, a change in the charge on the surface of the carrier, and the destruction of the carrier itself. The photoreaction also depends on the NIR laser output power and irradiation time, resulting in better control over drug/gene release. Photoisomerization induces conformational change of the isomer. Azobenzene derivatives have been widely used as a photoisomer. These UCNPs generate a rapid release of the drug/gene for cancer treatment. In addition, the UCNPs function as a UV source and allow spatial control of gene expression by blocking the irradiating area. Despite the development of drug/gene delivery systems using lanthanide-doped UCNPs, several problems remain. Drug/gene payload is important for effective therapy and successful gene expression. However, a constraint of the payload in nanocarriers limits the therapeutic efficacy of the drug/gene delivery system. Increasing the concentration of nanocarriers induces high toxicity in normal tissues. Thus, to overcome these limitations, further studies on drug/gene delivery systems that combine immunotherapy [90] or other therapies, such as photodynamic therapy (PDT) [91,92] or photothermal therapy (PTT) [93], have been reported. Researchers should also investigate and understand the leakage of drugs/genes from the cargo to prevent side effects. For example, Bazylińska et al. reported that the leakage of delivery cargo was prevented by encapsulating the drug and UCNPs using the double emulsion method, which forms hybrid nanocomposites [94,95]. Future research should be carried out to reduce the loss of therapeutic agents as well as the leakage of inorganic species by utilizing lanthanide-doped UCNPs as nanocarriers. In particular, dissociation of the carrier itself releases all components such as drug/gene molecules, lanthanide-doped UCNPs, and enveloping molecules. This release can interrupt metabolism and excretion behavior. Thus, future research should investigate the toxicity of the residue by long-term tracking for the biosafety of nanocomposites.
